# A *SCN1A* missense variant (c.4522T>A, p.(Tyr1508Asn) associated with genetic epilepsy with febrile seizures plus: clinical phenotype and genetic analysis of a Chinese pedigree

**DOI:** 10.3389/fgene.2026.1746234

**Published:** 2026-02-18

**Authors:** Xiao-Ling Li

**Affiliations:** Department of Pediatrics, Children’s Hospital Affiliated to Shandong University (Jinan Children’s Hospital), Jinan, China

**Keywords:** autosomal dominant inheritance, genetic epilepsy with febrile seizures plus, incomplete penetrance, missense variant, Nav1.1, SCN1A

## Abstract

Genetic epilepsy with febrile seizures plus (GEFS+, OMIM 604403) is a clinically and genetically heterogeneous epilepsy spectrum disorder characterized by phenotypic variability and complex inheritance patterns. The *SCN1A* gene (encoding the α1 subunit of the voltage-gated sodium channel Nav1.1) is the most frequently implicated driver, although variants in other sodium channel genes and synaptic pathway regulators have also been reported. Herein, we describe a GEFS + pedigree identified in clinical practice, with comprehensive genetic and phenotypic characterization. It should be noted that this family has been previously reported in a Chinese publication, and the present study provides further in-depth genetic and clinical analysis based on the original cohort. High-throughput sequencing of the proband followed by Sanger sequencing validation in family members identified a heterozygous missense variant in *SCN1A*: c.4522T>A p. (Tyr1508Asn). This variant was detected in five affected family members and one asymptomatic carrier. In accordance with the ACMG/AMP guidelines (2015) and ClinGen Epilepsy Sodium Channel Expert Panel specifications (Version 2.0.0), the variant was classified as a Variant of Uncertain Significance (VUS), given its absence from population databases (1000 Genomes, gnomAD, ESP6500) and clinical variant repositories (ClinVar, HGMD), as well as lack of prior literature reports. Co-segregation analysis confirmed consistent association between the variant and GEFS + spectrum phenotypes, and *in silico* predictions (PolyPhen-2, SIFT, VariantTaster) supported a deleterious effect on protein function. The inheritance pattern was consistent with autosomal dominant inheritance with incomplete penetrance. Structural analysis localized the variant to the intracellular D3-D4 linker of Nav1.1, a domain critical for fast channel inactivation, providing a plausible mechanistic basis for altered neuronal excitability. Our findings expand the spectrum of *SCN1A* variants associated with GEFS+ and highlight the importance of comprehensive pedigree analysis in deciphering the genetic basis of heterogeneous epilepsy syndromes. These data also provide clinically actionable insights for genetic counseling and precision medicine in affected families once the variant is proven to be pathogenic.

## 1 Introduction

Genetic epilepsy with febrile seizures plus (GEFS+) is a familial epilepsy spectrum disorder first delineated by Scheffer et al., in 1997, characterized by phenotypic heterogeneity ranging from simple febrile seizures (FS) persisting beyond 6 years of age to generalized or focal epilepsies with afebrile seizures. Classified as a primary channelopathy, GEFS+ is driven by variants in genes regulating neuronal excitability, with *SCN1A* being the most commonly implicated locus (Scheffer and Berkovic, 1997; Bonzanni et al., 2018). The *SCN1A* gene (NM_001165963.3) maps to chromosome 2q24.3, spans 81 kb, and comprises 29 exons encoding the α1 subunit of the voltage-gated sodium channel Nav1.1—an essential component of action potential generation and propagation in central nervous system (CNS) neurons, particularly GABAergic interneurons (Catterall et al., 2010). To date, over 1400 *SCN1A* variants have been linked to GEFS+, with genotype-phenotype correlations increasingly refined to distinguish mild (e.g., FS+) from severe (e.g., Dravet syndrome) phenotypes (Bryson and Petrou, 2023).

Despite extensive characterization of *SCN1A* variants, the genetic landscape of GEFS + remains incompletely defined, with variants continuously expanding the spectrum. Notably, phenotypic heterogeneity within GEFS + pedigrees—including variable penetrance and expressivity—poses challenges for genetic diagnosis and counseling. In this study, we report a *SCN1A* missense variant (c.4522T>A, p. (Tyr1508Asn) identified in a multi-generational GEFS + pedigree. We comprehensively analyze the clinical phenotypes, genetic co-segregation, and *in silico* functional predictions to evaluate the pathogenic potential of this variant. Our findings contribute to the understanding of *SCN1A*-mediated epilepsy and underscore the value of integrated clinical-genetic analysis in advancing precision medicine for epilepsy once the variant is proven to be pathogenic.

## 2 Materials and methods

To clarify the cause of the disease in this family, all participating individuals and parents/guardians of minors provided written informed consent. All procedures in this study were performed in accordance with the Helsinki Declaration.

### 2.1 Clinical phenotyping

Clinical data were collected through retrospective review of medical records, standardized interviews, and neurological examinations. Seizure phenotypes were classified according to the 2017 International League Against Epilepsy (ILAE) classification criteria (Fisher et al., 2017). Electroencephalographic (EEG) recordings (interictal video-EEG) and brain magnetic resonance imaging (MRI) were performed for all affected individuals. Neurodevelopmental and cognitive assessments were conducted using age-appropriate standardized tools.

### 2.2 Genomic DNA extraction

Peripheral blood samples (5 mL) were collected from 12 family members (6 affected, six unaffected). Genomic DNA was extracted using the Qiagen FlexiGene DNA Kit (Qiagen, Hilden, Germany) according to the manufacturer’s protocol. DNA quality and concentration were assessed using a NanoDrop 2000 UV spectrophotometer (Thermo Fisher Scientific, Waltham, MA, USA) and agarose gel electrophoresis.

### 2.3 High-throughput sequencing and variant filtering

A custom-designed next-generation sequencing (NGS) panel targeting 511 epilepsy-related genes (including *SCN1A, SCN2A, SCN8A, GABRG2*, etc.) was used for variant screening in the proband. Library preparation was performed using the Ion AmpliSeq Library Kit 2.0 (Thermo Fisher Scientific), and sequencing was carried out on the Ion Proton System (Thermo Fisher Scientific) with an average coverage depth of ≥100×. Raw sequencing data were processed using Ion Torrent Suite Software (v5.10), and variants were annotated using ANNOVAR. Filtering criteria included: 1. minor allele frequency (MAF) <0.01 in gnomAD, 1000 Genomes Project, and ESP6500 databases; 2. non-synonymous variants, splice-site variants, or indels; 3. variants predicted to be deleterious by at least two *in silico* tools (PolyPhen-2, SIFT, VariantTaster); 4. variants in genes previously associated with GEFS + or epilepsy.

### 2.4 Sanger sequencing validation and Co-segregation analysis

Primers flanking the candidate variant (*SCN1A* c.4522T>A) were designed using Primer3Plus (forward: 5′-GCC​CCA​TCC​CAA​GGT​TTA​CT-3′; reverse: 5′-TTT​GGG​GGT​GTT​TGT​CTT​CA-3′). PCR amplification was performed using AmpliTaq Gold 360 DNA Polymerase (Applied Biosystems, Foster City, CA, USA) under the following conditions: 95 °C for 10 min, 35 cycles of 95 °C for 30 s, 58 °C for 30 s, 72 °C for 30 s, and a final extension at 72 °C for 7 min. PCR products were purified using the QIAquick PCR Purification Kit (Qiagen) and sequenced on an ABI 3730 XL automated sequencer (Applied Biosystems). Sequences were aligned to the *SCN1A* reference sequence (NM_001165963.3) using SeqMan Pro (DNASTAR, Madison, WI, USA). Co-segregation analysis was performed by comparing variant status with clinical phenotypes in family members.

### 2.5 Bioinformatics and functional predictions

Sequence alignment was performed based on NCBI database information, and query candidate variant sites in HGMD, ESP6500, and gnomAD databases to exclude polymorphism. PolyPhen-2 (http://genetics.bwh.harvard.edu/pph2/), SIFT (https://sift.bii.a-star.edu.sg/), VariantTaster (https://www.varianttaster.org/), and MutationTaster2 (https://www.mutationtaster.org/) were used to predict the deleteriousness of candidate variants. Then, according to the American College of Medical Genetics and Genomics (ACMG) genetic variation classification standards and guidelines, the discovered variant sites were comprehensively analyzed to determine their pathogenicity (Richards et al., 2015). Variant interpretation was additionally performed using the ClinGen Epilepsy Sodium Channel Expert Panel specifications to the ACMG/AMP guidelines for *SCN1A* (Version 2.0.0). Evidence strength was adjusted according to *SCN1A*-specific recommendations, including conservative application of phenotype-based criteria and gene-specific weighting of segregation and population frequency evidence. This complementary framework was used to refine variant classification and to avoid overestimation of pathogenicity in the context of phenotypic heterogeneity.

## 3 Results

### 3.1 Clinical characteristics of the pedigree

**FIGURE 1:**
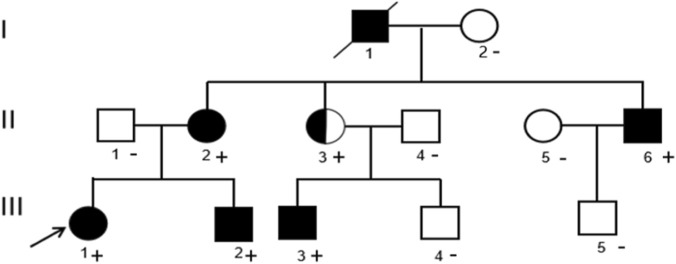
Pedigree of the family with GEFS+. Squares indicate males and circles indicate females. Filled symbols denote individuals with a clinical history within the GEFS + spectrum. Half-filled circle denotes the asymptomatic carrier with a heterozygous variant carrier. The proband is indicated by an arrow. Individuals with a “+” sign harbour the *SCN1A* c.4522T>A variant, whereas “–” indicates non-carriers. The maternal grandfather (I1) had a childhood history suggestive of febrile seizures but did not undergo genetic testing; therefore, his genotype is unknown and is indicated as not tested (NA).

The pedigree included 12 individuals across three generations, with six affected members (Figure 1). Clinical details of the proband and key family members are summarized in Table 1.

**TABLE 1:** Clinical data of proband (III1) and her younger brother (III2) in the pedigree.

Clinical features	Proband (III1)	Younger brother (III2)
Gender	Female	Male
Age	17-year-old	14-year-old
Phenotype	FS + phenotype with focal attacks	FS + phenotype with focal attacks
Age of febrile seizures	3 months	5 months
Age of non-febrile seizures	2–3 years old	1 year 5 months
Type of seizure	Generalized tonic clonic seizures and focal attack	Generalized tonic clonic seizures and focal attack
Duration of attack	1 min (generalized tonic clonic seizures) and 1–2 min (focal attack)	1–2 min (generalized tonic clonic seizures) and 1–2 min (focal attack)
Medical history, birth history, and developmental history	Unaffected	Unaffected
Physical examination	Unaffected	Unaffected
Psychomotor development	Unaffected	Unaffected
Neurological examination	Unaffected	Unaffected
EEG	Epileptiform discharge	Epileptiform discharge
Brain MRI	Unaffected	Unaffected
Antiepileptic drug response	Lamotrigine and levetiracetam	Sodium valproate and lamotrigine

The proband (III1) was a 17-year-old female with a history of seizures since infancy. Her first seizure occurred at 3 months of age and was classified as a febrile generalized tonic–clonic seizure (GTCS), characterized by loss of awareness, upward eye deviation, perioral cyanosis, and bilateral limb stiffening and clonic movements, lasting approximately 1 min and associated with a body temperature of 38 °C.

Between 1 and 2 years of age, she experienced recurrent febrile seizures (>10 episodes). At 2–3 years of age, she developed focal motor seizures with impaired awareness, manifested by unilateral eye deviation, left facial twitching, and clonic movements of the left upper limb, occasionally associated with fever. From 3 to 8 years of age, seizures occurred approximately 1–2 times per year, either febrile or afebrile. No seizures were reported between 9 and 13 years of age.

At 14 years of age, she experienced a recurrent afebrile focal seizure with impaired awareness, prompting medical evaluation. Interictal video-EEG demonstrated multifocal epileptiform discharges, including spike and spike–slow wave complexes predominantly over the bilateral frontal, frontotemporal, and Rolandic regions during sleep. Brain MRI, including hippocampal imaging, was unremarkable. Neurodevelopment and cognitive function were age-appropriate, with normal intelligence test results.

Treatment with lamotrigine was initiated and titrated to 125 mg twice daily. Due to a breakthrough focal seizure 6 months later, levetiracetam was added and titrated to 625 mg twice daily. The patient has remained seizure-free for approximately 3 years at the most recent follow-up.

The younger brother (III2) was a 14-year-old male with seizure onset at 5 months of age, presenting as a febrile generalized tonic–clonic seizure lasting 1–2 min. At approximately 17 months of age, he developed afebrile focal seizures with impaired awareness, characterized by behavioral arrest and oral automatisms without prominent limb motor involvement.

Between 6 and 8 years of age, seizure frequency increased to once every 2 weeks to once per month. Interictal video-EEG revealed asynchronous focal epileptiform discharges over the bilateral frontal and anterior temporal regions. Brain MRI and cognitive evaluation were unaffected. Sodium valproate was initiated and titrated to 0.25 g twice daily, followed by adjunctive lamotrigine (50 mg twice daily) due to incomplete seizure control. He subsequently achieved seizure freedom, with normalization of EEG findings on follow-up and normal developmental and cognitive outcomes.

Regarding other affected family members, the maternal grandfather (I1) had febrile seizures in childhood and is deceased. The mother (II2) experienced febrile seizures in early childhood followed by afebrile seizures, with remission after 10 years of age, consistent with febrile seizures plus (FS+). The uncle (II6) had febrile seizures in childhood. The cousin (III3) developed febrile seizures at approximately 1 year of age followed by afebrile focal seizures. Other family members were clinically unaffected.

### 3.2 Genetic findings

#### 3.2.1 Variant identification

High-throughput sequencing of the proband identified a heterozygous missense variant in *SCN1A*: c.4522T>A (NM_001165963.3), corresponding to a tyrosine-to-asparagine substitution at amino acid position 1508 p. (Tyr1508Asn). This variant was not present in the HGMD, ClinVar, or dbSNP databases, nor was it reported in prior literature. Population frequency databases (gnomAD, 1000 Genomes, ESP6500) showed no evidence of this variant in healthy controls (MAF = 0).

#### 3.2.2 Sanger sequencing and Co-segregation

**FIGURE 2:**
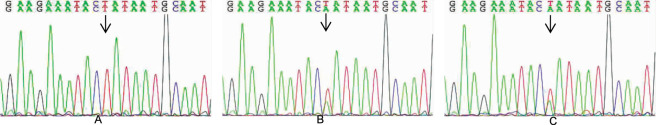
Sanger sequencing results of *SCN1A* gene in the GEFS + family. **(A)**: no variation was found; **(B)**: A heterozygous variant of c. 4522T>A in the proband; **(C)**: A heterozygous variation of c. 4522T>A in II2, II3, II6, III2, and III3.

Sanger sequencing (Figure 2)confirmed the variant in the proband and five family members (II2, II3, II6, III2, III3). Co-segregation analysis revealed that all affected individuals carried the variant, while asymptomatic non-carriers (I2, II1, II4, II5, III4, III5) had the wild-type genotype. The asymptomatic carrier (II3) was a heterozygous variant carrier. Due to the exclusion of untested deceased individual (I1) and the potential for the asymptomatic carrier (II3) to develop seizures later or have subclinical EEG abnormalities, a precise penetrance estimate cannot be reliably calculated.

#### 3.2.3 Pathogenicity classification

According to the ACMG/AMP guidelines and ClinGen recommendations, the *SCN1A* c.4522T>A variant was classified as a VUS based on the following evidence (PM2 + PP1 + PP3):

PM2: Absent from population databases (MAF <0.01).

PP1: Strong co-segregation with GEFS + phenotype in a multi-generational pedigree.

PP3: Deleterious predictions by multiple *in silico* tools (PolyPhen-2: Probably damaging, score = 0.998; SIFT: Deleterious, score = 0.01; VariantTaster: Disease-causing, score = 0.999).

#### 3.2.4 Amino acid conservation and structural analysis

**FIGURE 3:**
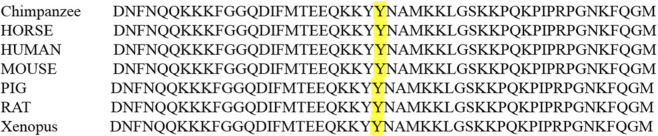
Cross-species conservation of the Tyr1508 residue in Nav1.1. The yellow highlights Tyr1508, which is conserved across all species analyzed.

Amino acid conservation analysis showed that Tyr1508 is highly conserved across humans, chimpanzees, horses, mice, and pigs (Figure 3), indicating functional importance. Structural modeling of the Nav1.1 D3-D4 linker was performed using SWISS-MODEL based on the crystal structure of the Nav1.7 sodium channel (a hypothetical model, given the lack of a resolved Nav1.1 structure). This model localized Tyr1508 to the intracellular D3-D4 linker of Nav1.1, a domain known to be critical for fast channel inactivation. The Tyr1508Asn substitution is predicted to alter the hydrophobicity and charge of the D3-D4 linker; however, without functional validation, any potential impact on channel inactivation gating remains speculative.

## 4 Discussion

### 4.1 Summary of key findings

In this study, we conducted a comprehensive clinical and genetic analysis of a multi-generational Chinese GEFS + pedigree, identifying a heterozygous missense variant *SCN1A* c.4522T>A p. (Tyr1508Asn) that mostly co-segregates with the disease phenotype. The variant is located in the functionally critical D3-D4 linker of Nav1.1, is absent from normal population databases, and is predicted to be deleterious by multiple *in silico* tools. According to ACMG/AMP guidelines combined with ClinGen specifications, the variant was classified as a VUS. The pedigree exhibits an autosomal dominant inheritance pattern with incomplete penetrance, and the clinical phenotypes are relatively mild, characterized by febrile seizures plus combined with focal seizures, with normal neurodevelopment and cognitive function in all affected individuals. In addition, this family has been reported in a previous Chinese study (Sun et al., 2021), and our research further supplements the genetic functional prediction and structural analysis data of this variant. Compared with our prior report on this pedigree, the present work provides critical supplementary data on variant pathogenicity prediction and clinical management insights, which significantly advances the understanding of this *SCN1A* variant-mediated GEFS + subtype.

### 4.2 Variant pathogenicity and mechanistic implications

GEFS+ is a genetically heterogeneous epilepsy spectrum disorder, with *SCN1A* variants accounting for the majority of cases (Polizzi et al., 2012). The *SCN1A* gene encodes the α1 subunit of Nav1.1, which is predominantly expressed in GABAergic interneurons—key regulators of neuronal excitability (Yu et al., 2006). Variants in *SCN1A* can lead to either loss-of-function (LOF) or gain-of-function (GOF) effects, with LOF variants typically associated with severe phenotypes (e.g., Dravet syndrome) and GOF variants linked to milder GEFS + phenotypes (Matricardi et al., 2023).

The *SCN1A* c.4522T>A variant maps to the intracellular D3-D4 linker of Nav1.1, a domain essential for fast channel inactivation (Cestèle and Catterall, 2000). This region contains conserved motifs that mediate the interaction between the D3-D4 linker and the channel pore, triggering rapid inactivation after depolarization (Kanai et al., 2006). Amino acid conservation analysis confirmed that Tyr1508 is highly conserved across species, supporting functional relevance. In silico predictions consistently classified the variant as deleterious, and hypothetical structural modeling (based on Nav1.7) suggests that the Tyr1508Asn substitution may disrupt the hydrophobic and charge properties of the D3-D4 linker. However, without direct functional validation (e.g., electrophysiological studies), any potential impact on inactivation gating remains speculative. This mechanism is consistent with prior reports of *SCN1A* variants in the D3-D4 linker associated with GEFS+ (Riva et al., 2021), highlighting the critical role of this domain in channel function.

Notably, the variant was classified as a VUS in strict adherence to ACMG/AMP guidelines. However, strong co-segregation with GEFS + phenotypes, absence from population databases, and consistent *in silico* predictions collectively support pathogenicity. Incomplete penetrance (observed in carrier II3) is a common feature of *SCN1A*-related epilepsy (Xu et al., 2015), likely influenced by modifying genetic factors, environmental triggers, and developmental stage-dependent neuronal excitability (Gotra et al., 2021).

### 4.3 Phenotypic heterogeneity in GEFS+

The pedigree exhibited phenotypic variability typical of GEFS+, ranging from simple febrile seizures (uncle II6) to FS+ with focal seizures (proband III1, brother III2, cousin III3). This heterogeneity is consistent with prior reports of *SCN1A* variants, where the same variant can manifest with diverse phenotypes within a family (Zhang et al., 2017). Factors contributing to phenotypic variability include variant location (e.g., core vs. non-core domains), functional impact (LOF vs. GOF), and genetic background (Brunklaus et al., 2022). In our study, the variant localized to the non-core D3-D4 linker, and the phenotypes were relatively mild (no epileptic encephalopathy, preserved cognition), aligning with the association between non-core domain variants and milder GEFS + phenotypes (Sheilabi et al., 2022). All affected individuals in the pedigree had normal developmental and cognitive outcomes, which was also consistent with the mild phenotypic spectrum of GEFS + associated with non-core *SCN1A* variants.

Notably, several affected individuals exhibited focal seizures with frontal/frontotemporal involvement, as evidenced by EEG findings. This expands the phenotypic spectrum of GEFS+, which is traditionally considered a generalized epilepsy syndrome (Scheffer and Berkovic, 2023). Recent studies have identified focal epilepsy phenotypes in GEFS + pedigrees with *SCN1A* variants (Antonini et al., 2019), suggesting that GEFS + should be recognized as a disorder of widespread neuronal excitability networks with variable regional expression. The frontal/frontotemporal involvement observed in our pedigree may reflect region-specific vulnerability of GABAergic interneurons expressing Nav1.1 (Tai et al., 2014).

### 4.4 Clinical implications

Our findings have important clinical implications for genetic counseling and precision treatment. The identification of the *SCN1A* c.4522T>A variant allows for targeted genetic testing of at-risk family members, enabling early diagnosis and intervention once classified as pathogenic with further evidence. For affected individuals, the relatively mild phenotype and response to sodium channel blockers (e.g., lamotrigine, sodium valproate) are consistent with observations in GEFS + cases associated with GOF *SCN1A* variants. However, it is critical to emphasize that clinical drug response alone is insufficient to infer channel function (GOF vs. LOF), and no functional data directly support a GOF effect for the c.4522T>A variant (Brunklaus et al., 2020). In contrast, LOF *SCN1A* variants (e.g., Dravet syndrome) may be refractory to sodium channel blockers and require alternative therapies targeting GABAergic function (Feng et al., 2023).

## 5 Limitations

This study has several limitations. First, functional studies (e.g., patch-clamp electrophysiology) were not performed to directly validate the variant’s impact on Nav1.1 channel function. Second, the pedigree size is relatively small, limiting the power to assess modifier genes. Third, the asymptomatic carrier (II3) was not evaluated with long-term EEG monitoring, leaving open the possibility of subclinical epileptiform activity. Future studies should include functional characterization of the variant and larger pedigree analysis to further clarify its pathogenicity and phenotypic correlates. Fourth, penetrance could not be reliably estimated due to untested deceased family members and the potential for delayed seizure onset or subclinical findings in the asymptomatic carrier.

## 6 Conclusion

We report a *SCN1A* missense variant (c.4522T>A, p. (Tyr1508Asn) in a GEFS + pedigree, expanding the spectrum of *SCN1A* variants associated with this disorder. The variant segregates with autosomal dominant inheritance with incomplete penetrance, and *in silico* analyses support a deleterious effect on Nav1.1 channel function. The phenotypic heterogeneity observed in the pedigree highlights the complex relationship between genotype and phenotype in *SCN1A*-related epilepsy. Our findings underscore the value of integrated clinical-genetic analysis in diagnosing heterogeneous epilepsy syndromes and provide actionable insights for genetic counseling and precision treatment once proven as pathogenic.

## Data Availability

The datasets generated and/or analyzed during the current study are available in the National Genomics Data Center (NGDC) repository under the accession number HRA013516 (https://ngdc.cncb.ac.cn/gsa-human/browse/HRA013516).
